# Epley’s maneuver in benign paroxysmal positional vertigo associated with Meniere’s disease

**DOI:** 10.1016/S1808-8694(15)30102-6

**Published:** 2015-10-19

**Authors:** Cristina Freitas Ganança, Heloisa Helena Caovilla, Juliana Maria Gazzola, Maurício Malavasi Ganança, Fernando Freitas Ganança

**Affiliations:** a Doctor om Science, post-graduate course on Human Communication Disorders, Sao Paulo Federal University - Paulista School of Medicine, UNIFESP-EPM.. Visiting professor in the Otoneurology Discipline of the Speech Therapy School, UNIFESP-EPM.; b Full professor in the Otoneurology Discipline, UNIFSP-EPM. Full professor in the Otoneurology Discipline, UNIFSP-EPM.; c Speech therapist, specialized in Gerontology, UNIFESP - EPM. Master’s degree in Science, post-graduate course on Otorhinolaryngology and Head & Neck Surgery, UNIFESP - EPM.; d Full professor of Otorhinolaryngology at UNIFESP - EPM. Coordinator of the postgraduate program for the master’s degree in The Science of Body Movement, Sao Paulo Bandeirante University, UNIBAN. Full professor of Otorhinolaryngology at UNIFESP - EPM.; e Doctor in Medicine, postgraduate course in Otorhinolaryngology and Head & Neck Surgery, UNIFESP - EPM. Associate professor in the Otoneurology Discipline, UNIFESP - EPM. Responsible for the Vestibular Rehabilitation Unit of the Otoneurology Discipline, UNIFESP - EPM. Professor of the postgraduate The Science of Body Movement program, UNIBAN.; f Doctor in Medicine, postgraduate course in Otorhinolaryngology and Head & Neck Surgery, UNIFESP - EPM, Associate professor in the Otoneurology Discipline, UNIFESP - EPM. Responsible for the Vestibular Rehabilitation Unit of the Otoneurology Discipline, UNIFESP - EPM. Professor of the postgraduate The Science of Body Movement program, UNIBAN. Sao Paulo Federal University - Paulista School of Medicine (UNIFESP-EPM).

**Keywords:** labyrinth, meniere’s disease, positional nystagmus, dizziness

## Abstract

The effects of Epley’s maneuver in benign paroxysmal positional vertigo (BPPV) associated with Menière’s disease are controversial.

**Aims:**

To evaluate the progression of positional vertigo and nystagmus after one or more of Epley’s maneuvers in BPPV associated with Menière’s disease, and the recurrence of BPPV.

**Method:**

a retrospective study of 62 patients with BPPV associated with Menière’s disease, that underwent Epley’s maneuver, and that were monitored during 12 months after elimination of positional nystagmus.

**Results:**

One Epley’s maneuver was required to eliminate positional nystagmus in 80.7% of the patients, two in 16.1%, and three in 3.2%; after elimination of nystagmus, positional vertigo was suppressed in 71.0% of the patients, improved in 27.4% and remained unaltered in 1.6%. Four weeks after elimination of positional nystagmus, all patients were asymptomatic. Recurrence of BPPV was seen in 19.4% of the cases, with elimination of the positional vertigo and nystagmus by means of the specific maneuver for the involved canal.

**Conclusions:**

In BPPV associated with Menière’s disease, vertigo and positioning nystagmus were eliminated with one, two or three Epley maneuvers. BPPV recurrence was resolved by using a specific maneuver for the affected canal.

## INTRODUCTION

In 1861, Prosper Ménière described a symptom triad composed of paroxystic events of tinnitus, hearing loss and vertigo with no central nervous system involvement. This condition received the name Ménière’s disease, being currently defined as an idiopathic endolymphatic hydrops syndrome.

According to the Committee on Hearing and Equilibrium guidelines for the diagnosis and evaluation of therapy in Ménière’s disease of the American Academy of Otolaryngology - Head and Neck Foundation, Inc.,^1^ Ménière’s disease is characterized by periodic crises. These are spontaneous and recurring vertigo events that vary from patient to patient and depend on the stage of the disease, lasting at least 20 minutes, and that usually are accompanied by nausea and/or vomiting; there is also spontaneous nystagmus (always present during a crisis), hearing loss, fullness of the ear and tinnitus in the affected side, and no loss of conscience.

Adler (in 1897) and Báràny (in 1921) described vertigo and paroxysmal nystagmus due to changes in the position of the head. Dix and Hallpike created a test to assess vertigo and positional nystagmus, and proposed the name benign paroxysmal positional vertigo (BPPV) for the condition that included the abovementioned signs.^2^

Dizziness upon head movements in BPPV would result from the undue presence of calcium carbonate particles resulting from fragmentation of canaliths from the macula acustica utriculi. Schuknecht^3^ and Schuknecht, and Ruby^4^ defined cupulolithiasis as the situation where these particles become deposited in the cupula of the posterior semicircular canal. Hall et al.^5^ proposed that these particles would float freely in the posterior semicircular canal (canalithiasis) rather than being fixed to the cupula. In 1983, Paparella and Mancini^6^ suggested that floating particles could cause labyrinthic hydrops due to obstruction of endolymphatic flow. In 1984, Paparella^7^ suggested that endolymphatic hydrops would cause positional vertigo in Ménière’s disease due to saccular distension up to the semicircular canals.

BPPV may be associated with Ménière’s disease or occur at any stage of this disease, usually during its stability phase, months or years after vertigo has regressed.^8^, ^9^, ^10^, ^11^, ^12^, ^13^, ^14^, ^15^, ^16^, ^17^, ^18^ On the other hand, Ménière’s disease may be considered as one of the causes of persisting vertigo in posterior semicircular canal BPPV patients.^19^

Epley^20^ described a canalith repositioning maneuver. A series of head movements would replace canalith fragments back in the utricle, where they would be absorbed or eliminated by the endolymphatic sac. This maneuver results in high improvement or cure rates and is frequently used in the treatment of BPPV that involves the anterior or posterior semicircular canals.^21^ If positional nystagmus persists, the maneuver should be repeated weekly until this ocular movement is abolished.

The maneuver has undergone various modifications throughout the years, all with similar success rates.^22^, ^23^, ^24^, ^25^

Recurrence is common and require repetition of the maneuver.^20^, ^26^, ^27^, ^28^, ^29^, ^30^, ^31^, ^32^, ^33^, ^34^, ^35^ BPPV-associated endolymphatic hydrops may lead to recurring vertigo and positional nystagmus after repositioning maneuvers, with persistence of symptoms in some patients.^12^, ^36^, ^37^, ^38^

The aim of this paper was to assess the clinical progression of vertigo and positional nystagmus after one or more Epley’s maneuvers in BPPV patients with Ménière’s disease and upon recurrence of BPPV.

## METHOD

This study was approved by the Research Ethics Committee of the institution in which the trial was done (protocol number 04821/04).

The sample series was selected from 1,946 new consecutive patient charts diagnosed as having BPPV and/or Ménière’s disease by an otorhinolaryngologist between January 1999 and July 2004. Inclusion criteria were patients diagnosed with BPPV and positional nystagmus evoked by the Dix-Hallpike test, a final diagnosis of Ménière’s disease in which Epley’s maneuver was used as the only treatment for BPPV and that were followed-up during twelve months after the Epley maneuver that abolished positional nystagmus.

The diagnosis of Ménière’s disease was based on criteria established by the Committee on Hearing and Equilibrium guidelines for the diagnosis and evaluation of therapy in Ménière’s disease of the American Academy of Otolaryngology-Head and Neck Foundation, Inc.1 Patients with uncontrolled Ménière’s disease used vestibular suppressant and antiemetics drugs during crises of vertigo.

The diagnosis of BPPV was based on reports of rotary dizziness upon changes in the position of the head, when moving to one or the other side while supine, when moving to the erect position or when looking upwards. Nausea and vomiting may be present or not, and vertigo and positional nystagmus are evoked by the Dix-Hallpike test. Between crises, there may be intermittent instability or other types of variable degree dizziness.^15^

The Dix-Hallpike test (lateral decubitus) was used to investigate positional nystagmus. The characteristics of positional nystagmus, when present, indicated the labyrinth and the semicircular canal that were involved and permitted the distinction between canalithiasis (nystagmus lasting less than a minute) and cupulolithiasis (nystagmus lasting more than one minute). Vertigo and/or nausea, latency, paroxysm and fatigue upon repetition of the procedure were signaled. Vertical upbeat and rotary positional nystagmus characterized posterior canal involvement; vertical downbeat and rotary positional nystagmus characterized anterior canal involvement; rotary clockwise or counterclockwise positional nystagmus indicated involvement of the vertical canal without separating the anterior and posterior canals, and geotropic or ageotropic horizontal positional nystagmus indicated lateral canal involvement.^15^

Sixty-two BPPV patients and a final diagnosis of Ménière’s disease fulfilled the inclusion criteria for this trial. Thirty-nine (62.9%) patients were female and 23 patients (37.1%) were male. Age ranged from 23 to 86 years (mean - 54.2 years). All subjects were Caucasian.

BPPV associated with Ménière’s disease patients underwent pure tone audiometry, voice audiometry, immitance testing and electrocochleography. Vestibular function was assessed by computerized nystagmography (Micromedical Technologies Inc., USA), which involved the following tests: calibration of ocular movements, investigation of positional/positioning nystagmus, spontaneous and semi-spontaneous nystagmus, fixed and randomized saccadic movements, pendular tracking, optokinetic nystagmus, self-rotation of the head and the air caloric test. A Dix-Hallpike test was done using video-Frenzel lenses (Neurograff Eletromedicina Ind. e Com. Ltda - EPT - Brazil) to define the involved canal and labyrinth.

The Epley maneuver was done in every case to reposition canalith particles from the semicircular canal back into the utricle. Patients were not prescribed restricted postures after the maneuver. Patients were evaluated by Dix-Hallpike testing one week later. If positional nystagmus persisted, Epley’s maneuver was repeated. This was done weekly, if needed, until positional nystagmus subsided. Patients were again evaluated by Dix-Hallpike testing four weeks after positional nystagmus had been abolished.^39^

Patients were informed about the possibility of recurrence of BPPV signs and symptoms, which would require them to return for reassessment and retreatment. Upon recurrence of vertigo and positional nystagmus on Dix-Hallpike testing, Epley’s maneuver was repeated in cases with vertical semicircular canal involvement; cases where the lateral semicircular canal was involved were treated with Lempert’s and Tiel-Wilck’s40 positional maneuver.

Findings were analyzed statistically, starting with the descriptive analysis of data. The chi-square test was used in analyzing the association between frequencies of a two-category sample, followed by Yates’s correction. The chi-square test was also used in checking the association of frequencies in a three-category sample. The chi-square (x^2^) test was used in evaluating the association between two independent samples in a contingency table. Fisher’s exact test was used in identifying the association between variables in the contingency table in which 25% of cells had occurrences below five. The Kaplan-Meier test was used in analyzing BPPV recurrence prediction where a group of subjects was assessed in given periods (days or months) at unequally spaced intervals. The significance level for statistical tests was 5% (α = 0.05).

## RESULTS

Charts from 1,946 new consecutive patients with a diagnostic hypothesis of BPPV and/or Ménière’s disease between January 1999 and July 2004 were analyzed. BPPV was diagnosed in 1033 cases (53.1%), Ménière’s disease was diagnosed in 841 cases (43.2%) and BPPV associated with Ménière’s disease was diagnosed in 72 cases (3.7%). Ten BPPV associated with Ménière’s disease patients were excluded due to loss of follow-up.

Sixty-two patients with BPPV and a final diagnosis of Ménière’s disease fulfilled the inclusion criteria for this study. BPPV started before Ménière’s disease in one patient (1.6%); the onset of BPPV coincided with that of Ménière’s disease in four patients (6.5%); the onset of BPPV was after stabilization of the clinical picture of Ménière’s disease 57 patients (91.9%).

On the Dix-Hallpike test, all 62 patients showed vertigo and positional nystagmus with latency, paroxism, duration below one minute and fatigue upon repeating the procedure. Positional nystagmus suggested unilateral involvement of the posterior semicircular canal in 62 patients, as follows: vertical upbeat rotatory counterclockwise positional nystagmus in the right lateral decubitus characterizing right labyrinth involvement in 37 cases (59.7%), and vertical upbeat rotatory clockwise positional nystagmus in the left lateral decubitus characterizing left labyrinth involvement in 25 cases (40.3%).

Ménière’s disease was unilateral in 40 cases (64.5%) and bilateral in 22 cases (35.5%). In unilateral cases, right labyrinth involvement occurred in 20 patients (50%) and left labyrinth involvement was found in 20 patients (50%).

Table 1 shows the number of cases of BPPV associated with Ménière’s disease according to the labyrinth that was involved. In right labyrinth BPPV Ménière’s disease was ipsilateral in 20 patients (54.1%), contralateral in one patient (2.7%) and bilateral in 16 patients (43.2%). In left labyrinth BPPV, Ménière’s disease was ipsilateral in 19 patients (76%) and bilateral in six patients (24%). There was a significant association between involvement of both labyrinths in the two diseases (p<0.001). BPPV tended to be prevalent in the right labyrinth in patients with bilateral involvement of Ménière’s disease (p=0.055).

**Table 1:** Number of cases of benign paroxysmal positional vertigo associated with Ménière’s disease, according to the labyrinth that was involved

		Ménière’s disease			
Condition		Right labyrinth	Left labyrinth	Bilateral	Total
BPPV	Right labyrinth	20	1	16	37
	Left labyrinth	0	19	6	25

	Total	20	20	22	62

**Key:**BPPV - benign paroxysmal positional vertigo

Test - Fisher’s exact test

Significance - p<0.001

Table 2 shows that one session of Epley’s maneuver was needed to eliminate positional nystagmus in BPPV in 50 patients (80.7%), two sessions of Epley’s maneuver was needed in 10 patients (16.1%) and three sessions of Epley’s maneuver was needed in two patients (3.2%). The need for one session of Epley’s maneuver to abolish positional nystagmus was much more frequent than the need for two or three sessions of Epley’s maneuver (p<0.001).

**Table 2:** Number and percentage of patients with benign paroxysmal positional vertigo associated with Ménière’s disease according to the number of sessions of Epley’s maneuver needed to abolish positional nystagmus.

Number of Epley’s maneuvers	Patients with BPPV and Ménière’s disease	
	Number	%
One	50	80,7
Two	10	16,1
Three	2	3,2

Total	62	100,0

**Key:**BPPV - benign paroxysmal positional vertigo

Test - chi-square

Significance - p<0.001

The progression of positional vertigo on the Dix-Hallpike test is shown on Table 3. After positional nystagmus was abolished by Epley’s maneuver, 44 cases (71%) remained asymptomatic, 17 cases (27.4%) reported improvement and one case (1.6%) was unaltered. Asymptomatic cases were significantly more prevalent compared to improved and unaltered cases (p<0.001).

**Table 3:** Number and percentage of patients with benign paroxysmal positional vertigo associated with Ménière’s disease according to the progression of positional vertigo in the Dix-Hallpike test one week following Epley’s maneuver that resulted in extinction of positional nystagmus.

Progression of positional vertigo in the Dix-Hallpike test	Patients with BPPV and Ménière’s disease	
	Number	%
Asymptomatic	44	71,0
Improved	17	27,4
Unaltered	1	1,6

Total	62	100,0

**Key:**BPPV - benign paroxysmal positional vertigo

Test - chi-square

Significance - p<0.001

Four week after the extinction of positional nystagmus, all 62 patients were asymptomatic and showed no positional vertigo on the Dix-Hallpike test.

BPPV relapsed in 12 cases (19.4%) as seen on follow-up 12 months after the maneuver that abolished positional nystagmus; these patients presented vertigo and positional nystagmus on the Dix-Hallpike test. Absence of recurrence of BPPV was significantly more frequent than the occurrence of relapses (p<0.001).

BPPV relapsed in two patients (3.2%) at two months, in one patient (1.6%) at three months, in two patients (3.2%) at six months, in two patients (3.2%) at eight months, in one patient (1.6%) at nine months, in one patient (1.6%) at 10 months, in two patients (3.2%) at 11 months and in one patient (1.6%) at 12 months after the maneuver that abolished positional nystagmus.

BPPV recurrence was observed in the same labyrinth as before in ten cases (83.3%) and in the opposite labyrinth in two cases (16.7%). The posterior semicircular canal was involved in eight patients, the anterior semicircular canal was involved in one patient and the lateral semicircular canal was involved in three patients. Recurring vertigo and positional nystagmus were abolished after one session of Epley’s maneuver in cases where the anterior or posterior semicircular canals were involved, and after Lempert’s and Tiel-Wilck’s positional maneuver in cases where the lateral semicircular canal was involved.

**Figure 1:**
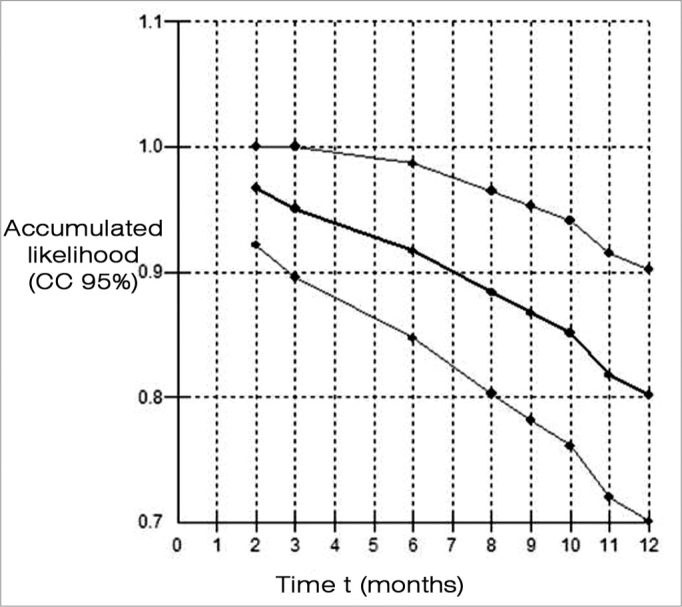
Kaplan-Meier estimator for the curve of non-recurring cases of benign paroxysmal positional vertigo up to 12 months in 62 patients with Ménière’s disease.

Figure 1 shows the Kaplan-Meier estimator for the curve of non-recurring cases. The accumulated BPPV recurrence chance was 3.3% at two months, 5% at three months, 8.3% at six months, 11.6% at eight months, 13.3% at nine months, 14.9% at 10 months, 18.3% at 11 months and 19.9% at 12 months.

## DISCUSSION

BPPV is a clinical entity with a high incidence in the world population; it may be considered as the most frequent vestibular condition.^15^, ^41^, ^42^ This disease is benign and may be treated by specific vestibular rehabilitation procedures, such as canalith repositioning procedures, vestibular suppressant drugs or surgery in refractory cases to medical treatments.^15^, ^37^, ^42^

In the initial survey for this study, 1,105 patients were diagnosed as having BPPV, of which 1,033 cases (53.1% of the total number of charts that were evaluated) had BPPV exclusively and 72 cases (6.5%) had BPPV associated with Ménière’s disease. Seventy-two (7.9%) of 913 patients (46.9% of the total number of charts that were evaluated) diagnosed as having Ménière’s disease had BPPV. Ten BPPV patients associated with Ménière’s disease were excluded from our series due to loss of follow-up. BPPV may be associated with Ménière’s disease or with other otoneurological diseases.^9^, ^10^, ^11^, ^12^, ^13^, ^38^ The occurrence of Ménière’s disease in BPPV patients has been cited in other papers as representing 0.6%,^13^ 0.9%,^8^ 2.1%,^9^ 3.2%^16^ and 29.8%^10^ of cases. BPPV in patients with Ménière’s disease has been diagnosed by various authors in 5.5%,^36^ 10.0%,^37^ 11.5%,^12^ 14.1%^38^ and 44.0%^11^ of cases.

In this study 39 patients (62.9%) with BPPV associated with Ménière’s disease were females aged between 23 and 86 years (mean age was 54.2 years), similar to findings in papers on BPPV associated with Ménière’s disease.^10^, ^36^, ^37^, ^38^.

In our series, the onset of BPPV occurred after stabilization of Ménière’s disease in 57 cases (91.9%). This finding is comparable to that in a study in which the onset of BPPV occurered after Ménière’s disease in all cases.36 Some authors have reported that the onset of BPPV occurred after Ménière’s disease in 33.3% of cases, and that it was not possible to establish the initial entity in 64.5% of cases.10 According to other authors, the onset of both diseases was simultaneous in 55.6% of cases.37

Involvement of the posterior semicircular canal by canalithiasis occurred in all the 62 patients with Ménière’s disease and BPPV that were evaluated in this study, which is similar to findings by other authors.^9^, ^37^, ^38^ Localization in the posterior semicircular canal appears to favor the migration of canaliths that become detached from the utricle.^31^

BPPV was unilateral in all of the cases of this study, occurring in the same labyrinth that was affected by unilateral Ménière’s disease in 39 patients (62.9%) and contralateral to unilateral Ménière’s disease in only one case (1.6%). These findings agree with published data in the literature where most cases of BPPV were unilateral and the affected side was the same as that involved by Ménière’s disease.10,36-38 There was no mention in the literature we consulted about the occurrence of BPPV in the ear not affected by Ménière’s disease. In our cases of bilateral Ménière’s disease, BPPV tended to present in the right labyrinth.

Ménière’s disease is a possible etiology for BPPV.^7^, ^12^, ^13^ This study demonstrated that most of the cases of unilateral BPPV occurred after the onset of ipsilateral Ménière’s disease, reinforcing the idea that the latter condition might alter the otolytic organ and be responsible for loosening particles that then migrate to the semicircular canals, resulting in canalithiasis. Floating canaliths might induce endolymphatic hydrops by obstructing longitudinal endolymphatic flow.^7^ In cases where BPPV preceded Ménière’s disease, floating canalith fragments might induce hydrops by mechanically obstructing endolymphatic flow and by altering the absorption of endolymph.^6^

A single session of Epley’s maneuver was sufficient to eliminate positional nystagmus on the Dix-Hallpike test in 80.7% of patients diagnosed as having BPPV associated with Ménière’s disease. This finding is significantly higher than the need for using two or three sessions of Epley’s maneuver for similar results. One week after performing one to three sessions of Epley’s maneuver 98.4% of cases became asymptomatic or improved substantially from positional vertigo. Asymptomatic cases were more frequent than improved or unaltered cases. Four weeks after abolishing positional nystagmus, all 62 patients were asymptomatic and presented no positional vertigo on the Dix-Hallpike test. Notwithstanding the extinction of positional nystagmus on the Dix-Hallpike test, persistence of positional vertigo in BPPV after the repositioning procedure may be due to remaining canalith particles in the posterior canal. These particles would be insufficient to flex the cupula and produce nystagmus, but would suffice to induce vertigo.^17^

The efficacy of Epley’s maneuver in the treatment of BPPV patients has been underlined in the literature.^16^, ^14^, ^20^, ^21^, ^26^, ^28^, ^30^, ^31^, ^32^, ^33^, ^34^, ^35^, ^42^, ^43^, ^44^, ^45^ Only a few authors with smaller series compared to this series have investigated Epley’s maneuver in patients diagnosed as having BPPV associated with Ménière’s disease. Two patients remained with positional vertigo and four patients remained with nystagmus as evidenced by the Dix-Hallpike test.46 Elimination of positional nystagmus with the persistence of dizziness or mild positional unbalance was seen in 32.1% of the affected labyrinths.^12^ Repeated repositioning procedures resulted in temporary improvements in one patient, elimination of symptoms in another patient and persistence of vertigo e do nystagmus.^37^

Some authors^12^, ^36^, ^37^ have reported increased difficulties in resolving BPPV associated with Ménière’s disease compared to our findings for cases in which the onset of BPPV occurred after that of Ménière’s disease. Improvement of vertigo and elimination of positional nystagmus, however, was seen in 83.3% of cases of BPPV associated with Ménière’s disease after a single repositioning procedure,^38^ which may be considered as similar to our findings in this study.

The success of treating BPPV secondary to some other labyrinthic disease by Epley’s maneuver, including Ménière’s disease cases, is usually inferior compared to cases of idiopathic BPPV.^16^

After successful treatment of BPPV patients by using Epley’s maneuver, recurrence rates of signs and symptoms have been quoted at 6.6%^32^ to 12.0% of cases after six months,^31^ 15%^27^ to 30%^20^, ^26^ of cases at one year, 50% of cases at 40 months,^28^ 37% of cases 60 months after treatment, 29 and 43% of cases at two years.^33^ Recurrence was seen in 50% of cases after a ten-year follow-up period. Recurrences occurred in 80% of cases during the first year following the treatment.^35^

The one-year follow-up of our patients diagnosed as having BPPV associated with Ménière’s disease showed recurrence of vertigo and positional nystagmus in 19.4% of cases two to 12 months after the repositioning maneuver that abolished positional nystagmus. The number of cases with no recurrence of BPPV was significantly higher than the number of recurring cases. Studies on patients diagnosed as having Ménière’s disease associated with BPPV have shown a 50% recurrence rate^12^, ^38^ between on average 8.3 months to one year.

The recurrence of BPPV in this study was in the same side and the same semicircular canal that had been involved previously. Only two cases recurred in the opposite side, as has been reported in the literature.^36^, ^37^, ^38^ Recurrence in a semicircular canal other than the previously involved canal was seen in four cases, of which three were in the lateral canal and one in the anterior canal. We found no references of BPPV recurrence in a different semicircular canal in patients with Ménière’s disease. We also found no explanation for recurrence in the opposite side in patients diagnosed as having BPPV with Ménière’s disease. Ménière’s disease might be responsible for BPPV recurrence in the opposite labyrinth.

Treatment with a single repositioning procedure was successful in all of the recurring BPPV cases in this study. Patients reported partial improvement from BPPV after repositioning procedures, with persistence of positional vertigo that was tolerable in routine activities. Some authors have noted that Ménière’s disease might predispose the patient to intractable BPPV, as all of their patients had on average two to three BPPV recurrences within one year, possibly due to permanent structural damage to the macula or the membranous labyrinth.^36^

In our study, we found that within one year following the session of Epley’s maneuver that abolished positional nystagmus, the accumulated chance of BPPV recurrence varied from 3.3% after two months to 19.9% after 12 months. No information was found in the literature for comparison with this finding.

Findings in this study demonstrated that Epley’s maneuver was effective in the treatment of BPPV and its relapses in patients with Ménière’s disease. It is essential to inform patients with a diagnosis of Ménière’s disease associated with BPPV about possible recurrences of positional vertigo and the need for further treatment. These patients should be under long-term monitoring.

## CONCLUSION

After one, two or three sessions of Epley’s maneuver in BPPV patients associated with Ménière’s disease, the findings were as follows:

1) elimination of positional nystagmus;
2) elimination of positioning vertigo in up to four weeks after extinction of positional nystagmus, and
3) follow-up at 12 months showed BPPV recurrence cases in which vertigo and positional nystagmus were abolished after one maneuver aimed at the diseased semicircular canal.
